# Comparison of Oleo- *vs* Petro-Sourcing of Fatty Alcohols via Cradle-to-Gate Life Cycle Assessment

**DOI:** 10.1007/s11743-016-1867-y

**Published:** 2016-09-12

**Authors:** Jignesh Shah, Erdem Arslan, John Cirucci, Julie O’Brien, Dave Moss

**Affiliations:** 1 Air Products, 7201 Hamilton Blvd, Allentown, PA 18195 USA; 2 Spatial Analytics, Lehigh Valley, PA 18078 USA; 3 Air Products Performance Materials Division, 7201 Hamilton Blvd, Allentown, PA 18195 USA

**Keywords:** Life cycle assessment, Alcohol ethoxylates, Fatty alcohol, Palm kernel oil, Oleochemicals, Cradle-to-gate analysis, Greenhouse gases, Environmental impacts, Malaysian palm, LCA

## Abstract

Alcohol ethoxylates surfactants are produced via ethoxylation of fatty alcohol (FA) with ethylene oxide. The source of FA could be either palm kernel oil (PKO) or petrochemicals. The study aimed to compare the potential environmental impacts for PKO-derived FA (PKO-FA) and petrochemicals-derived FA (petro-FA). Cradle-to-gate life cycle assessment has been performed for this purpose because it enables understanding of the impacts across the life cycle and impact categories. The results show that petro-FA has overall lower average greenhouse gas (GHG) emissions (~2.97 kg CO_2_e) compared to PKO-FA (~5.27 kg CO_2_e). (1) The practices in land use change for palm plantations, (2) end-of-life treatment for palm oil mill wastewater effluent and (3) end-of-life treatment for empty fruit bunches are the three determining factors for the environmental impacts of PKO-FA. For petro-FA, *n*-olefin production, ethylene production and thermal energy production are the main factors. We found the judicious decisions on land use change, effluent treatment and solid waste treatment are key to making PKO-FA environmentally sustainable. The sensitivity results show the broad distribution for PKO-FA due to varying practices in palm cultivation. PKO-FA has higher impacts on average for 12 out of 18 impact categories evaluated. For the base case, when accounted for uncertainty and sensitivity analyses results, the study finds that marine eutrophication, agricultural land occupation, natural land occupation, fossil depletion, particulate matter formation, and water depletion are affected by the sourcing decision. The sourcing of FA involves trade-offs and depends on the specific practices through the PKO life cycle from an environmental impact perspective.

**Electronic supplementary material:**

The online version of this article (doi:10.1007/s11743-016-1867-y) contains supplementary material, which is available to authorized users.

## Introduction

Non-ionic surfactants are used in many products such as “detergents, cleaners, degreasers, dry cleaning aids, petroleum dispersants, emulsifiers, wetting agents, adhesives, agrochemicals, including indoor pesticides, cosmetics, paper and textile processing formulations, prewash spotters, metalworking fluids, oilfield chemicals, paints and coatings, and dust control agents” [1].

Nonylphenol ethoxylates (NPE) are popular non-ionic surfactants “due to their effectiveness, economy and ease of handling and formulating” [2]. However, NPE are highly toxic to aquatic organisms [1, 2] and degrade into nonylphenol (NP), which “is persistent in the aquatic environment, moderately bioaccumulative, and extremely toxic to aquatic organisms” [1]. Due to these concerns, the US Environmental Protection Agency (EPA) and detergents manufacturers cooperated to eliminate their use in household laundry detergents [3]. Also, EPA has laid out action plan to address widespread use in large quantities in industrial laundry detergents under the Toxic Substances Control Act [3]. Due to higher biodegradability and unobjectionable aquatic toxicity profiles of the degradation products, alcohol ethoxylates (AE) are used to replace NPE [2]. AE are also nonionic surfactants that are produced via ethoxylation of fatty alcohol (FA) with ethylene oxide (EO). This involves condensation of polyethylene glycol ether groups on FA chains. Depending on the FA structure and number of polyether units, the physical and chemical properties of AE vary [4]. When the chain length of FA ranges in C_9_–C_16_, the properties are suitable for detergents production [4] for industrial and institutional cleaning products including hard surface cleaners and laundry detergents.

In addition to these product stewardship practices, sustainability minded companies are also evaluating the environmental impact of their operations, as well as the burdens from the other phases of product life cycle, including raw material sourcing. With respective to raw material sourcing, a bio-based value chain is often assumed to have less environment impact, at least from greenhouse gases (GHG) emissions perspective. For AE producers, the source of FA could be either bio-based oleochemicals (oleo-FA) or petrochemicals (petro-FA). These AE with like structures (linearity-wise and chain lengths) are readily biodegradable independent of alcohol feedstock and their aquatic toxicities are function of FA chain length, branching and amount of ethoxylation [5]. These similarities in the environmental performance at the product’s use and end-of-life phases do not capture differences in environmental impacts during the raw material production. The detailed understanding of the raw material requirements, energy consumption, waste generations and disposal, and emissions, along with the resulting impacts on the environment, is important for sustainability-minded AE consumers and other supply chain participants.

Such an understanding could be gained through a life cycle assessment (LCA) approach as it allows incorporation of all relevant life cycle stages along with diverse types of environmental impacts. LCA is the comprehensive evaluation of the process in a cradle-to-grave, cradle-to-gate or gate-to-gate fashion to understand the environmental aspects of a product or a service. LCA study involves understanding the assessment goal and scope; estimating the amount of raw materials and energy input, waste generated, and emissions from the process for all the relevant life cycle stages (Life Cycle Inventory, LCI); translating LCI results to understand and evaluate the potential environmental impacts (Life Cycle Inventory Assessment, LCIA); and formulating conclusions and recommendations based on the results. LCA has been used since the 1960s and its application for surfactants started with developing of LCI [6–8]. These early studies compiled data on the natural resources consumed, wastes generated, and emissions for then-industry practices for AE production from both petrochemical and oleochemical feedstocks. However, the impacts from land transformation for palm plantation were not covered and the scope was limited to LCI due to lack of agreed-upon LCIA methods. The results from these LCI studies did not find any scientific basis for any single feedstock source to be environmentally superior [6, 8] as “benefits in one direction (e.g., renewability) are offset by liabilities in another (intensive land-use requirements)” [6]. LCA studies for detergents since then have been based on the results of these earlier studies and are for the products with AE and FA as ingredients such as that by Kapur et al. 2012 [9]. In 2007, the ‘ecoinvent data v2.0’ project [4] updated the LCI results from the earlier studies with land use, transportation and infrastructure information. However, again the LCIA and conclusions steps were not done. The LCA results from production of palm derived oil, which is used for FA production, have been published [10–13]. The scopes of these studies vary from evaluating the impacts of oil from palm fruits and/or palm kernels [11, 12] to evaluating the various practices for palm oil mill operations [10, 13]. Overall, there has been no LCA study with LCIA results evaluating impacts of feedstocks for FA production.

This study aims to contribute towards this gap and presents the findings for understanding the relative environmental performances of sourcing FA from petrochemical and palm kernel oil (PKO) feedstocks. These findings are expected to contribute to the discussions towards such an understanding rather than a final conclusion as such.

## Experimental Methods

While LCA has been around since 1960s, it was not widely adopted until the early 1990s. Currently, LCA is guided by international standards (ISO 14040 to ISO 14044), which have proposed the framework for conducting an LCA study [14]. As per this framework, LCA involves four iterative steps: (1) Goal and scope definition, (2) Life cycle inventory analysis (LCI), (3) Life cycle impact assessment (LCIA) and (4) interpretation. The intended and expected applications of the results help define the goal and scope. The results and findings of LCI are checked with goal and scope to decide whether goal and scope should be modified or additional effort should be spent on LCI step. Similarly, LCIA results and findings are evaluated against previous two steps. The results from LCI and LCIA steps are interpreted with respect to goal and scope and for robustness. The results of this fourth step are evaluated against the other three steps for any modification or additional efforts. This standard methodology was used for this study and the detailed descriptions could be found in ISO 14040 through ISO 14044.

The goal of this study was to create an understanding of the relative environmental impacts for selecting between petro-FA and PKO-FA^1^ for use in AE production. A comparative LCA study was performed because it allows simplification of the scope to the dissimilar parts of each process.

FA are predominantly linear and monohydric aliphatic alcohols with chain lengths between C_6_ and C_22_ [4]. Despite the differences in FA sourcing, “the chemical and physical properties of the final product [AE] are similar for all three pathways [petrochemical, PKO, coconut oil], provided their carbon chain length and ethoxylate distribution is similar” [4]. However, depending on the catalyst and olefins used, not all petro-FA produced via hydroformylation technology compete with PKO-FA [15]. The scope of this study has been limited to FA that could be used interchangeably irrespective of feedstocks.

**Fig. 1:**
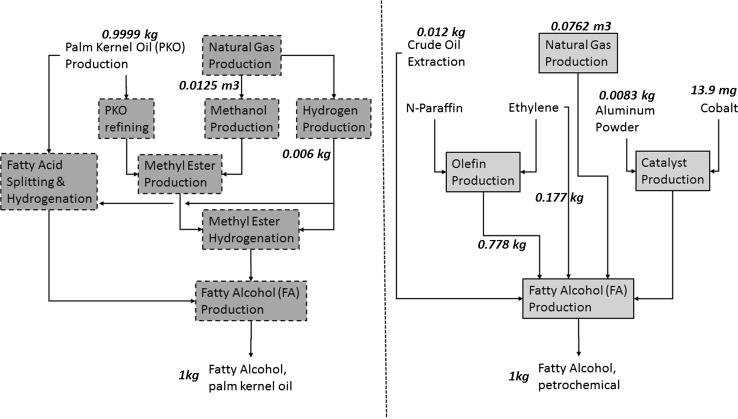
Major process steps for the various fatty alcohol production pathways. Adapted from [4]

Once a FA is produced and delivered, the environmental impacts are similar irrespective of FA sourcing decision. Likewise, FA sourcing decisions do not impact AE use and AE end-of-life treatment. Hence, a cradle-to-gate type boundary has been selected for this study (see Fig. 1) and all the results have been converted to one kg of FA delivered to AE production facility. In LCA terms, the functional unit for this study is one kg of FA delivered to AE production facility in Gulf Coast region of United States (US). The study has been performed through modeling in SimaPro 8.0 software for LCA studies.

The modeling in LCA requires input of quantities of raw materials and energy required, waste generated and emissions from the FA production process. Similarly, the production and distribution of these raw materials and their utilization generate the environmental impacts. For PKO-FA, the impacts are also generated from the land transformation for palm plantations and from the waste generated during the palm oil mill operation. For all these processes and the impacts including the production and delivery of FA, the data used for this study are secondary and literature data.

### Petro-FA

The petro-FA can be produced either via Ziegler process using hydrogenated catalyst triethylaluminium for alkylation of ethylene or via Oxo process using syngas for hydroformylation of long chain olefins [4]. The Ziegler process involves hydrogenation, ethylation, growth reaction, oxidation and hydrolysis of ethylene over Aluminum powder in presence of hydrocarbon solvent. While solvent is recovered, Aluminum exits the system as co-product alumina hydrate. Alkanes and oxygen-containing compounds are formed as byproducts [16]. Oxo process involves catalytic hydroformylation, catalyst recovery, catalytic hydrogenation of intermediate aldehydes and alcohol distillation of olefins and synthesis gas. While the catalyst consumption is minimal here, there are isomerization byproducts formed during hydroformylation, which are taken out during distillation as bottom heavies and overhead lights [16].

EcoInvent 3.0 (EI3.0) dataset for petro-FA production (“Fatty alcohol {RoW}| production, petrochemical | Alloc Def, U”) includes inputs and emissions reflecting a mix of 82 % of fatty alcohols produced with the Oxo process and 18 % produced by the Ziegler process. This dataset has taken the material inputs (ethylene, *n*-olefin, natural gas and crude oil), energy inputs (heat and electricity), solid waste generation, emissions to air, emissions to water, and impacts from transportation from literature sources while estimated water consumption and infrastructure. The disposal of solid waste is included via the process for municipal solid waste incineration and the effluent is captured through emissions to water. Further, it must be noted that this ‘gate-to-gate’ process also includes the impacts from some upstream processes (see Petro-upstream section). Table 1 summarizes the gate-to-gate LCI for petro-FA production.

**Table 1:** ‘Gate-to-gate’ LCI for fatty alcohol production and delivery

	Petro-FA	PKO-FA	Data sources
Raw materials/feedstocks			
*N*-olefins	0.778 kg		Petro: literature value by EI3.0
Ethylene	0.177 kg		Petro: literature value by EI3.0
Natural gas	0.0762 m^3^	0.0125 m^3^	Petro: literature value by EI3.0; PKO: Adapted data from ECOSOL study by EI3.0
Crude oil	0.012 kg		Petro: literature value by EI3.0
Aluminum powder	0.0083 kg		Petro: estimated by author based on stoichiometry
Cobalt	1.39E-5 kg		Petro: Estimated by author based on stoichiometry
Palm kernel oil		0.9999 kg	PKO: estimated by EI3.0 based on ECOSOL study data
Hydrogen		0.006 kg	PKO: estimated by EI3.0 based on ECOSOL study data
Utilities and infrastructure			
Water (cooling)	0.024 m^3^	0.024 m^3^	Assumed by EI3.0 based on literature for a large chemical plant
Water (process)	0.006 m^3^	0.006 m^3^	Estimated by EI3.0 as 25 % of the cooling water amount
Heat	5.81 MJ	11.83 MJ	PKO: estimated by EI3.0 based on ECOSOL study data
Electricity	0.166 kWh	0.161 kWh	
Transportation (road)	0.06 tkm	0.06 tkm	Updated by author based on the geographic scope
Transportation (ocean)	0.0 tkm	20 tkm	Updated by author based on the geographic scope
Chemical factory	4E-10 plant	4E-10 plant	Estimated by EI3.0 from a large chemical plant
Byproducts			
Alumina	−0.0157 kg		Petro: Estimated by author based on stoichiometry
Solid waste			
Solid waste incinerated	0.00339 kg	0.0195 kg	Petro: literature value by EI3.0; PKO: Adapted data from ECOSOL study by EI3.0
Direct air emissions			
Carbon dioxide, fossil		6.1E-5 kg	PKO: estimated by EI3.0 based on ECOSOL study data
Non-methane volatile organic compounds		2.05E-4 kg	PKO: estimated by EI3.0 based on ECOSOL study data
Particulates, >10 µm	7.95E-6 kg		Petro: literature value by EI3.0
Particulates, 2.5–10 µm	1.07E-5 kg		Petro: literature value by EI3.0
Particulates, <2.5 µm	6.21E-6 kg		Petro: literature value by EI3.0
Nitrogen oxides	2.06E-4 kg		Petro: literature value by EI3.0
Ammonia	1.68E-5 kg		Petro: literature value by EI3.0
Water vapor	0.0105 kg	0.0105 kg	EI3.0 calculated value based on literature values and expert opinion
Sulfur dioxide	7.5E-4 kg		Petro: literature value by EI3.0
Carbon monoxide, fossil	1.41E-4 kg		Petro: literature value by EI3.0
Direct water emissions			
Wastewater effluent	0.0195 m^3^	0.0195 m^3^	EI3.0 calculated value based on literature values and expert opinion
Ammonium, ion	8.42E-6 kg		Petro: literature value by EI3.0
COD, chemical oxygen demand	1.2E-4 kg	1.33E-3 kg	Petro: calculated by EI3.0 as 2*BOD5; PKO: Adapted data from ECOSOL study by EI3.0
TOC, total organic carbon	4.45E-5 kg	4.93E-4 kg	Calculated by EI3.0 as COD/2.7 where COD is measured in g O_2_

While this EI3.0 petro-FA process is fairly comprehensive, the dataset is for technology in mid-1990s as practiced in Europe for the “Rest of World” (RoW) region. The transportation impacts are based on the average distances and the commodity flow surveys. It is unclear how the various byproducts and wastes streams are handled. In order to address these concerns, the original dataset from EI3.0 has been modified as per the following discussions.

### Petro-FA Upstream

Since the dataset is for a different region other than the US, there could be an effect on the results due to potential differences in the production process, difference in the electricity grid mix and heat generation mix for FA production, the differences in the transportation and so on. The dataset for petro-FA in EI3.0 for RoW region was generated via modification of the Europe region by updating the electricity grid mixes, transportation impacts and heat generation impacts. The dataset description is said to be valid from 1995 till 2013.

The approach used by EI3.0 has been adapted to obtain a dataset for the US gulf coast region. The electricity grid mix was updated to Southeastern Electric Reliability Council (SERC). The heat generation process used in the petro-FA dataset and the raw material *n*-olefin production dataset were changed to “Heat, central or small-scale, natural gas {SERC}| heat production, natural gas, at boiler condensing modulating <100 kW | Alloc Def, U”. This dataset for heat was derived from that for Switzerland (“Heat, central or small-scale, natural gas {CH}| heat production, natural gas, at boiler condensing modulating <100 kW | Alloc Def, U”) provided by SimaPro 8.0 by updating the natural gas source to be from North America, the emissions profile for CO_2_, CO, CH_4_, N_2_O, NO_X_, SO_2_, lead, mercury and PM_10_ as per NREL data [17] and electricity to SERC grid. Based on the AE production facility location, it is expected that the natural gas produced in US is delivered via pipeline to the FA manufacturing facility in the Gulf Coast region of US for petro-FA. This petro-FA is expected to be delivered via truck to AE manufacturing facility. The transportation distances for FA production facility to AE production facility are estimated to be ~60 km for the respective plants located in US Gulf Coast region. The transportation is expected to be entirely via diesel combination trucks.

The crude oil and natural gas resources require some land transformation and occupation for the drilling and other auxiliary processes. Further, the chemical plants for the processing of these and the intermediates also require land use. For the latter, the dataset “Chemical factory, organics {GLO}| market for | Alloc Def, U” has been included by datasets in EI3.0. For the former, the impacts are included in the datasets as well [4]. However, the impacts from the process steps are not split up due to the format of data availability. Hence, the impacts from land use change and the waste from drilling operation are accounted for in this process rather than via separate upstream process. Overall, the cradle-to-gate impacts are included.

### Petro-FA Catalysts

Both Ziegler and Oxo routes use catalysts. EI3.0 process for petro-FA does not have aluminum powder and a hydrocarbon solvent as input and alumina hydrate as co-product applicable for the Ziegler process. Alumina hydrate has value in catalytic processes, in ceramics and other industrial applications. Since the solvent is recovered and recycled, exclusion is reasonable. With aluminum powder and alumina hydrate, there is no indication that the corresponding impacts are included. Hence, a separate dataset was created and included to account for the upstream (Raw material to Gate) impacts.

SimaPro 8.0 doesn’t have any dataset for aluminum powder used for Ziegler process. This dataset, hence, was modeled with “Aluminium, primary, ingot {GLO}| market for | Alloc Def, U” EI3.0 dataset as a starting point. Aluminum powder is expected to be produced via gas atomization of molten ingot. The energy needed for melting (*H*_melt_) is the primary consideration here and was estimated in J/g as per following equation from [18]

1 $$ H_{\text{melt}} = C_{\text{s}} \times \left( {T_{\text{m}} - T_{0} } \right) + H_{\text{f}} + C_{\text{l}} \times (T_{\text{p}} - T_{\text{m}} ) $$

where *C*_s_ is the weight specific heat for solid Aluminum (0.91 J/g/°C), *T*_m_ is the melting temperature of Al = 600 °C, *T*_0_ is the starting temperature (25 °C assumed), *H*_f_ is the heat of fusion for Al (10,580 J/mol [18]), *C*_l_ is the weight specific heat of the molten Al (1.086 J/g/°C), *T*_p_ is the pouring temperature (1700 °C [19]). 120 % multiplication factor was used as per [18] to account for energy losses. The resulting energy is estimated to be about 90 % of total energy need as additional energy is needed in holding furnace [49]. Argon gas is expected to be used here. The volume of Argon for atomization of Ti6Al4 V from the literature [20] was adjusted for Al atomization [18]. The cooling water consumption was estimated as per process specification for “Industrial Metal Powder Aluminum Powder Production Line” [19].

As per Zeigler reaction stoichiometry, 1 mol of Al yields 3 mol of FA translating into 0.05 kg Al for 1 kg FA. Similarly, one mole of alumina hydrate is produced per mole of Al translating into 0.11 kg alumina hydrate per kg FA produced. The credits from Alumina co-product is as per dataset “Aluminium oxide {GLO}| market for | Alloc Def, U” for EI3.0.

For the Oxo process, the cobalt carbonyl (HCo(CO)_4_) catalysts are used in 0.1–1.0 wt% concentration.^2^ The loss for catalyst is estimated to be <1 % [23]. This translates into 0.343–3.43 mg of Co need per kg of product. The impacts for the catalyst were accounted through “Cobalt {GLO}| market for | Alloc Def, U” EI3.0 dataset.

### Petro-FA Process Technology

EI3.0 dataset for petro-FA is based on 18 % Ziegler route production and 82 % Oxo route production as per mid-1990s data. The current validity of this split was confirmed.

In 2000, about 1.68 million metric tonnes of fatty alcohol was produced with 40 % being petro-FA [24]. The petro-FA production capacity in 2000 were estimated to 0.273 million tonnes for Shell’s Geismar LA plant [24], 0.17 million tonnes for BASF’s oxo-alcohol plant in Ludwigshafen [24], 0.10 million tonnes increase capacity for Sasol’s oxo-alcohol [24] and 0.06 million tonnes for BP [25]. These translate into 0.603 million tonnes of oxo-alcohol capacity, which would account for 90 % of petro-FA produced in 2000. In 2010, 90 % capacity utilization was estimated [26]. Considering new capacity installation between 2000 and 2005 (see discussion for 2005 below), this utilization rate should be reasonable and at such utilization rate, accounted oxo-alcohols formed about 81 % of petro-FA in 2000. It must be noted that base oxo-chemical capacity of Sasol is not accounted here due to lack of information. So, the split between oxo-route and Ziegler-route holds till 2000 and any small perturbation in this split does not significantly change the overall environmental impact of petro-route.

In 2005, 2.2–2.5 million tonnes of fatty alcohol production capacity has been estimated with 50 % being petro-FA [26]. The petro-FA production capacity in 2005 were estimated to 0.49 million tonnes for Shell [25, 27], 0.31 million tonnes for BASF [27], 0.25 million tonnes capacity for Sasol’s oxo-alcohol [28, 29] and 0.0 million tonnes for BP^3^ [25]. These translate into 1.05 million tonnes of oxo-alcohol capacity, which would form 86 % of petro-FA capacity in 2005. Similar to 2000, the split between oxo-route and Ziegler-route holds till 2005. In 2012, the total fatty alcohol capacity has been estimated to be 3.35 million tonnes with all of 0.8 million tonnes of capacity increase for oleo-FA [26]. Again, the split between oxo-route and Ziegler-route holds till 2012.

### Petro-FA Process Byproducts

Both Ziegler route and Oxo route generate byproducts.

With the Oxo route, ~5 wt% of olefin feed gets converted to byproducts [22], 5–10 wt% of olefins remains unreacted [30, 31] and ~2 mol% of aldehydes being unreacted during hydrogenation [32]. These unreacted materials and byproducts are distilled out with unreacted olefins recycled to hydroformylation stage and unreacted aldehydes to hydrogenation stage [33]. The light ends are either used as high grade fuel or blend stream for gasoline [33, 34]. The heavy ends are either used as fuel or solvents [31, 33]. It is difficult to tell whether the existing EI3.0 dataset for petro-FA has assigned the byproducts as fuel substitute, co-products, mixture or not at all. Considering the small amount of concern here, the choice here is not expected to impact the final conclusion within the scope of this study.

With the Ziegler route, besides for alumina hydrate discussed in the catalysts section, a small percentage of olefins form alkanes and oxygen-containing compounds as byproducts [16]. During the fractionation of crude alcohol formed, these byproducts could either be separated as waste or become part of certain blends. Considering the small amount of concern, the choice is not expected to impact the final conclusion within the scope of this study. Further, the EI3.0 dataset for petro-FA does account for some wastes that get incinerated.

### PKO-FA

The oleo-FA can be produced either via fatty acid splitting route (“Lurgi direct hydrogenation” of fatty acids obtained by splitting triglycerides from crude vegetable oil) or transesterification route (hydrogenation of methyl esters obtained by transesterification of crude or refined vegetable oil) [4]. In this study, the scope for the raw materials is limited to PKO and the production route limited to fatty acid splitting, esterification of refined PKO and esterification of crude PKO processes. In 2005, ~44 % of global palm fruit were produced in Malaysia (MY) [11]. Hence, PKO is expected to be produced in Malaysia and delivered via truck to FA manufacturing facility in Malaysia. The resulting PKO-FA is then via combination of truck-ship-truck to AE manufacturing facility in the US.

EI3.0 dataset for PKO-FA production (“Fatty alcohol {RoW}| production, from palm kernel oil | Alloc Def, U”) includes inputs and emissions reflecting a technology mix of 27 % produced from fatty acid splitting, 56 % produced from methyl ester on the basis of crude vegetable oil and 17 % from methyl ester out of refined oil. This dataset includes the material and energy inputs (methanol, palm kernel oil, natural gas and hydrogen), emissions to air and water, transportation and production of waste. Both processes (Fatty Acid splitting and transesterification) yield ~40 wt% of PKO as glycerin. Fatty Acid splitting also yields some short-chain (C_8_–C_10_) fatty alcohols, which could be estimated to be ~5 wt% based on the average fatty acid composition for PKO [35]. For transesterification process, when the PKO is refined first, ~5 wt% of PKO results in fatty acid distillate [36]. All these by-products have value. The mass-based allocations made in EI3.0 datasets for these multioutput processes were kept. Further, it must be noted that this ‘gate-to-gate’ process also includes the impacts from some upstream processes (see PKO-upstream section). Table 1 summarizes the gate-to-gate LCI for PKO-FA production.

While this EI3.0 PKO-FA process is fairly comprehensive, the dataset is for the “Rest of World” (RoW) region with palm kernel oil sourced globally. For this study, PKO sourcing region of interest is Malaysia. Similar to petro-FA dataset in EI3.0, the transportation impacts are based on the average distances and the commodity flow surveys. In order to address these concerns, the original dataset from EI3.0 has been modified as per the following discussions.

### PKO-FA Upstream Datasets

The dataset for PKO-FA in EI3.0 for RoW region was generated via modification of the one for Europe by updating the electricity grid mixes, transportation impacts and heat generation impacts. Such dataset is said to be valid from 2011 till 2013 as per dataset description. This approach used by EI3.0 has been adapted here to obtain a dataset for Malaysia.

Since FA is produced at a facility in Malaysia, the electricity grid mix from EI3.0 dataset for PKO-FA is updated from global electricity mix to “Electricity, medium voltage {MY}| market for | Alloc Def, U”. The heat generation process used in the PKO-FA dataset was changed to “Heat, central or small-scale, natural gas {MY}| heat production, natural gas, at boiler condensing modulating <100 kW | Alloc Def, U”. This dataset for heat was derived from that for Switzerland (“Heat, central or small-scale, natural gas {CH}| heat production, natural gas, at boiler condensing modulating <100 kW | Alloc Def, U”) provided by SimaPro 8.0 by updating the natural gas source to be from “Rest of World” (due to lack of dataset for natural gas from MY) and electricity to MY grid. The transportation distances for FA production facility to AE production facility is estimated to be ~20,000 km for the transoceanic shipment from Malaysia to US Gulf coast via Panama. Also, the truck transportation of ~60 km is expected between the ports and production facilities. Here, the transportation impacts for the various feedstock materials and waste are considered in terms of distance to be traveled, the amount to be transported, and the mode of transportation. The capital goods and infrastructure needed for the production and transportation are only considered when already covered in EI3.0 and other datasets used in SimaPro 8.0.

For methanol production related impacts, the natural gas resources (from which methanol is derived) were used. Such natural gas resources require some land transformation and occupation for the drilling and other auxiliary processes. Further, the chemical plants for the processing of these and the intermediates also require land use. For the latter, the dataset “Chemical factory, organics {GLO}| market for | Alloc Def, U” has been included by datasets in EI3.0. For the former, the impacts are included in the datasets as well [4]. However, the impacts from the process steps are not split up due to the format of data availability. Hence, the impacts from land use change and the waste from drilling operation are accounted for in this process rather than via separate upstream process. Overall, the cradle-to-gate impacts are included.

**Fig. 2:**
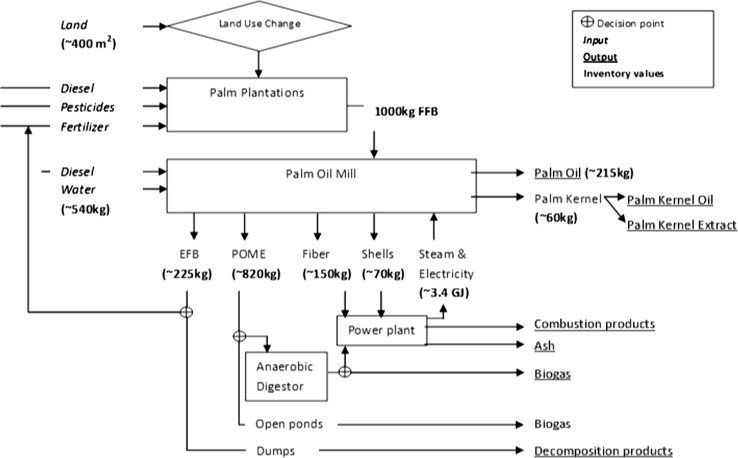
Process steps for production of Palm Kernel Oil and average inputs and outputs (adapted from 10) About 400 m^2^ of land, diesel, pesticides, fertilizer and water are major inputs required to produce 1000 kg Fresh Fruit Bunches (FFB). Processing of thus produced FFB in Palm Oil Mill takes diesel and about 540 kg water. Also, about 3.4 GJ of energy in form of steam and electricity is needed, which is obtained through use of shells and fibers generated from the oil mill. About 150 kg of fiber and about 70 kg of shells are generated. Also, about 225 kg of empty fruit bunches (EFB) are generated, which are either mulched for fertilizer substitute for plantations or dumped to rot. About 829 kg of POME (effluent from palm oil mill) is generated and disposed of either via untreated river discharge or anaerobic digestion of BOD present. The methane from digestion could be used for energy generation, flared or vented. Of the remaining mass of inputs to the oil mill, about 215 kg becomes palm oil, about 27 kg becomes palm kernel oil (PKO) and rest about 33 kg becomes palm kernel extract (PKE) used as animal feed substitute. The treatment options for EFB and POME are the decision points for the individual plantations and shown via + symbol

In the existing EI3.0 dataset for PKO-FA, the raw material production datasets are for global region. The PKO production dataset was updated so that 100 % of PKO was sourced from Malaysia. PKO is a co-product of palm oil production from the palm fruits produced as 10–40 kg Fresh Fruit Bunches (FFB) on the palm trees [11]. The growing of these trees (and, hence, the production of palm fruits) require the transformation of land for palm plantations initially, and then occupation of this land [11]. The palm plantations yield on average ~25 tonnes FFB per hectare [11]. FFB consists of ~22 wt% empty fruit bunches (EFB), ~65 wt% fleshy mesocarp (pulp) and ~13 wt% in an endosperm (seed) in the fruit (Palm Kernel). The mesocarp provides Palm Oil (PO) while the seed provides Palm Kernel Oil (PKO). The yield is ~22 wt% of FFB results in PO, ~2.7 wt% in PKO and ~3.3 wt% in Palm Kernel Extract (PKE). The kernel is protected by a wooden endocarp or Palm Kernel Shell (PKS). The solid waste left after the extraction of oils, including the fibers in pulp (~15 wt%), PKS (~7 wt%) and EFB, could be re-used as fuel substitute in energy generation and as fertilizer substitute via mulching. There is also liquid waste generated from the wastewater produced during the processing in oil mills. This wastewater effluent, termed Palm Oil Mill Effluent (POME), contains hydrocarbon contents (water and ~28 wt% of FFB) that could be repurposed for fertilizer substitute or recovered for fuel substitute. There are also air emissions due to the fuel combustion for energy generation. These various aspects for PKO can be seen in Fig. 2. The economic allocation with allocation factor of 17.3 % to PKO as used in EI3.0 dataset was used to allocate the impacts and credits between PO and PKO. Even though the allocation values are based on 2006 prices, they were found to be valid based on the prices in 2014 [37, 38]. EI3.0 dataset for palm plantations accounts for the benefits/impacts from growing palm trees such use of CO_2_ from air.

EI3.0 dataset for palm kernel oil production accounts the end-of-life treatments for the EFB, PKS and PKF via their combustion for supplying steam and electricity for the oil mills. The literature survey indicates that only PKS and PKF are used as fuel [39] and provide more than sufficient energy for oil mills [39]. EFB has been cited as “a resource which has huge potential to be used for power generation, currently not being utilized” [39]. The treatment of POME in EI3.0 is as standard wastewater. Recent publications [40] cited methane leaks from palm oil wastewater as a climate concern. In order to account for these differences, existing EI3.0 dataset for palm kernel oil was updated and new datasets were created to capture these differences in waste treatment.

The screening level analysis suggested that PKO raw material is the single largest GHG contributor for PKO-FA accounting for the differences in GHG emissions compared to petro-FA. Hence, PKO production (including palm plantations and oil mills) processes were evaluated in details as discussed below.

### POME Treatment Options

The end-of-life treatment for the POME could be discharge into a river without any treatment, after anaerobic digestion of organics with venting of thus-produced methane, after anaerobic digestion of organics with flaring of methane produced, or after anaerobic digestion of organics with recovery of methane for energy. The end-of-life treatment for the POME is expected to impact the pollution from the discharge of organics, generation of methane and CO_2_ from organics discharge and from the discharge of nitrogen compounds. The organics emissions were estimated as per the following equation:

2 $$ {\text{OM}}_{\text{POME}}^{\text{emitted}} {\text{ = COD}}_{\text{POME}} $$

where COD_POME_ is the Chemical Oxygen Demand generated from discharge of organics in POME. The methane emissions were estimated as per the following equation:

3 $$ {\text{CH4}}_{\text{POME}}^{\text{emitted}} = {\text{COD}}_{\text{POME}} \times B_{0} \times {\text{CF}}_{\text{CH4}} $$

where *B*_0_ is the methane producing capacity from the organics discharged and CF_CH4_ is the correction factor to the methane production capacity based on the conditions into which organics are discharged. The nitrogen emissions were estimated as per the following equation:

4 $$ {\text{N}}_{\text{POME}}^{\text{emitted}} {\text{ = Ncontent}}_{\text{POME}} $$

where Ncontent_POME_ is the nitrogen content discharge in the river depending on whether POME is treated or not. The values used for the parameters in Eqs. (2)–(4) for the various end-of-life treatment scenarios as per Achten et al. 2010 [41] can be found in Table S1. The emissions avoided from use of captured biogas for heat were estimated via EI3.0 dataset for cogen (“Heat, at cogen 50kWe lean burn, allocation heat/CH U”). The emissions from flaring of captured biogas were estimated via EI3.0 dataset for Refinery gas flaring (“Refinery gas, burned in flare/GLO U”). The literature survey showed that the lack of demand for thermal energy and limited/missing access to the national electricity grid has resulted in only ~30 % of palm oil mills recycling POME [10, 42] and only 5 % of POME gets treated to generate biogas for heat production with the rest 95 % being treated to just vent the generated biogas as shown in Table S2 [43]. Hence, a sensitivity analysis was done with the various disposal options for POME.

### PKS & PKF Treatment

EI3.0 dataset for palm kernel oil production accounts the direct emissions from the combustion of PKS and PKF via modified ‘wood chips, burned in a cogen 6400 kWth process. The modification of the ‘wood chip’ process accounts for the differences in dry matter, carbon content and the energy content. In this original EI3.0 approach, about 12.8 MJ of energy is generated per kg of oil produced. Of this, about 8.2 MJ is obtained from PKS and PKF. Approximately 7.84 MJ energy requirement for oil mill operation is reported in literature [10, 39, 44–46]. This aligns with Abdullah and Sulaiman 2013 observation that PKF and PKS are sufficient to meet oil mill’s energy demand [39]. Hence, the combustion impacts from original EI3.0 dataset were reduced to produce only 8.2 MJ. While this might be slightly in excess, it is expected that excess PKF & PKS will be treated the same way for convenience.

### EFB Treatment Options

For Malaysia, 75 % of the time EFB is expected to be mulched and for the rest 25 % dumped to rot [43]. EFB rotting was based on the modeling done by Stichnothe and Schuchardt (2011) [10], which is based on IPCC guideline for estimating GHG emissions from parks and garden waste. For rest of the nutrients, 50 % leaching was assumed, except 90 % leaching for potassium based on Rabumi (1998) [47]. The initial nutrient values for EFB are shown in Table S3. For mulching, the dataset in Simapro 8.0 was used and the fertilizer value of the mulch was estimated based on literature data [44, 47–50] shown in Table S4. The mulching process was captured through EI3.0 dataset (“Mulching {GLO}| market for | Alloc Def, U”) and about 10 km trucking was assumed [44]. The recycling of EFB was similar to the POME recycling situation [10, 42]. Hence, the sensitivity analysis was done with the various disposal options for EFB to evaluate the impacts from 100 % (ideal) and 0 % (the worst case) mulching.

### Land Use Change Options

As discussed earlier, palm plantations require land. This needed land could be from secondary forests, existing cropland, primary tropical forest and/or peatland. The transformation of this land from its current primary function to another function constitutes a land use change (LUC). LUC has significant environmental implications due to biodiversity impacts, water flow impacts, soil erosion impacts, GHG emissions and such. With respect to GHG emissions, the impacts are due to disruption or destruction of carbon stocks in above ground biomass (AGB), below ground biomass (BGB), soil and dead organic matter (DOM) along with N_2_O stock for peatland [10]. “The impact of LUC depends on various factors such as cultivation methods, type of soil and climatic conditions” [10].

For this study, the land transformation from the existing cropland, primary tropical forest, peatlands and secondary forest have been evaluated with the base case being the current practices in Malaysia (Table S5). The literature survey indicated that “peatland makes up 12 % of the SE [South East] Asian land area but accounts for 25 % of current deforestation. Out of 270,000 km^2^ of peatland, 120,000 km^2^ (45 %) are currently deforested and mostly drained” [10] presenting a case for sensitivity with LUC. The impacts from indirect LUC^4^ have been excluded from this study similar to earlier studies [41, 51] as we did not find any studies with the required data or methodology.

Currently, EI3.0 has datasets for existing cropland (“Palm fruit bunch {MY}| production | Alloc Def, U”) and primary tropical forest (“Palm fruit bunch {MY}| production, on land recently transformed | Alloc Def, U”) in SimaPro 8.0. The new datasets were created in SimaPro 8.0 for various types of land transformation by adjusting the value for “Carbon, organic, in soil or biomass stock” in primary tropical forest dataset. The values for secondary forest were derived by taking the ratio of primary forest and secondary forest in respective EI3.0 datasets for other regions. For peatland covered with primary forest, the values were assumed to be same as those for primary forest with extra BGB that gets drained. The value for BGB for peatlands were updated based on literature surveys [45, 51]. These adjustments (see Table S5) for the LUC, which are not covered in the datasets in SimaPro 8.0, only captures the GHG emissions related differences.

Assumptions in relation to the data:

1. Existing EI3.0 dataset for PKO production does not include negative impacts from EFB rotting, fertilizer use reduction from EFB mulching (benefit) and POME’s CH_4_ emissions.
2. No transportation losses.
3. Impacts from LUC are spread over 20 years.

The inventory data collected for petro-FA and PKO-FA along with assumptions capture the quantity of inputs and outputs of materials, energy, waste and emissions for the respective process. This inventory was converted to the functional unit basis (1 kg of FA delivered to AE production site). Such inventory (LCI) was modeled into SimaPro 8.0 software and then subjected to impacts assessment to understand and evaluate the potential environmental impacts by converting LCI results into impacts and aggregating these impacts within the same impact category to obtain the characterized results. ReCiPe Midpoint (H) method as implemented in Simapro 8.0 was used to obtain the characterized results for 18 impact categories. This method by default neither credits for CO_2_ intake from air for plant growth nor penalizes for biogenic CO_2_ emissions. In biofuel processes, since the CO_2_ intake by the plants is ultimately released with energy back into the atmosphere within a short timeframe, the credits and emissions balances out to carbon neutrality. However, in this case, the carbon intake is stored in the chemical products for a long time and may not necessarily be released as CO_2_ like combustion processes. Further, since FA end-of-life is out of scope in this cradle-to-gate study, CO_2_ intake needs to be included. Hence, the method was updated to account for CO_2_ intake and biogenic CO_2_ emissions. Also, the biogenic methane GWP factor was changed from 22 to 25 kg CO_2_e. 

The contribution analyses of the characterized results were performed to understand the hotspot areas of impacts and identify the key factors. For these key factors, the sensitivity analyses were performed to evaluate the various scenarios of LUC, POME end-of-life treatment and EFB end-of-life treatment. The uncertainty analyses were performed for both FA sourcing options for the base case via Monte Carlo sampling to understand the distribution. The number of samplings used was 1000 for both options.

## Results

Both petrochemical feedstocks and PKO feedstocks used for FA production are co-products and have other uses. For example, only a fraction of crude oil is used as feedstocks for FA production. This crude oil, which is derived as co-products, could be used for other applications such as energy. Similarly, PKO is co-product from PO production and could be used for other applications such as biodiesel or cooking oil. In other words, both feedstocks are part of large and complex supply chain.

**Fig. 3:**
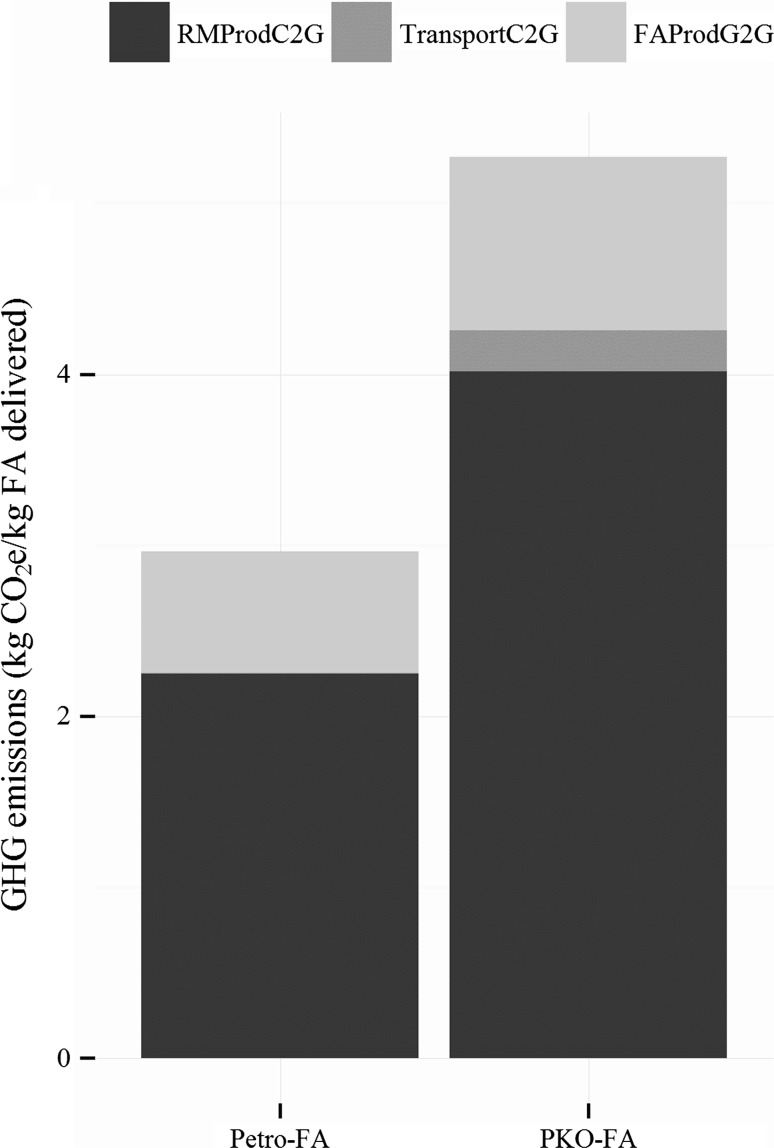
Contributions of various life cycle phases to the Life cycle GHG emissions for PKO-FA (fatty alcohol produced from palm kernel oil feedstock) and petro-FA (fatty alcohol produced from petrochemical feedstock) are shown in kg CO_2e_/kg FA delivered. The various life cycle phases shown here are RMProdC2G, Transport C2G and FAProdG2G. RMProdC2G includes the raw material production (includes the impacts from the transformation of inputs from nature via various intermediate products into the raw material delivered to the fatty alcohol (FA) production site. RMC2G also includes any transportation required till RM reaches the FA production site. FAProdG2G includes the production of FA from raw materials (e.g., PKO and *n*-olefins and ethylene). TransportC2G includes the transportation of FA produced from the FA production site to Alcohol Ethoxylates (AE) production site. Irrespective of the feedstocks, RMProdC2G is the most impactful phase for the boundary covered in this study. It accounts for 60+ and 75+ % of the life cycle GHG emissions for PKO-FA and petro-FA, respectively

For each kg of FA delivered, on a cradle-to-gate basis, petro-FA has ~2.97 kg CO_2_e emissions on average, which are ~55 % of ~5.27 kg CO_2_e emissions for PKO-FA on average (see Fig. 3). For petro-FA, the production of various raw materials contributed ~79 % of the total ~2.97 kg CO_2_e/kg FA delivered. Another ~21 % are from FA production and <0.2 % from transportation of raw material for FA production and of FA for AE production. Almost all of the GHG emissions during petro-FA production are from the combustion of natural gas in the US. Of climate change impacts from raw materials, ~70 % is from *n*-olefins production and delivery, ~10 % from ethylene production and delivery, ~10 % from upstream fuel production/combustion, ~8 % from catalysts (aluminum powder and cobalt), and the ~2 % remaining from solid waste handling and chemical plant infrastructure. For PKO-FA, the production of various raw materials contributes ~83 % of the total ~5.27 kg CO_2_e/kg FA delivered. Another ~12 % are from FA production and ~5 % from transportation of raw materials for FA production and of FA for AE production. Almost all of the GHG emissions during PKO-FA production are from the combustion of natural gas in MY. Due to lower GHG intensity for the combustion of natural gas in MY, the production GHG emissions are similar to petro-FA despite twice the thermal heat consumption. Of climate change impacts from raw materials, ~91 % are from PKO production, ~7 % from upstream fuel production/combustion, and the rest split between those from hydrogen production and delivery, chemical plant infrastructure and those from municipal solid waste.

**Fig. 4:**
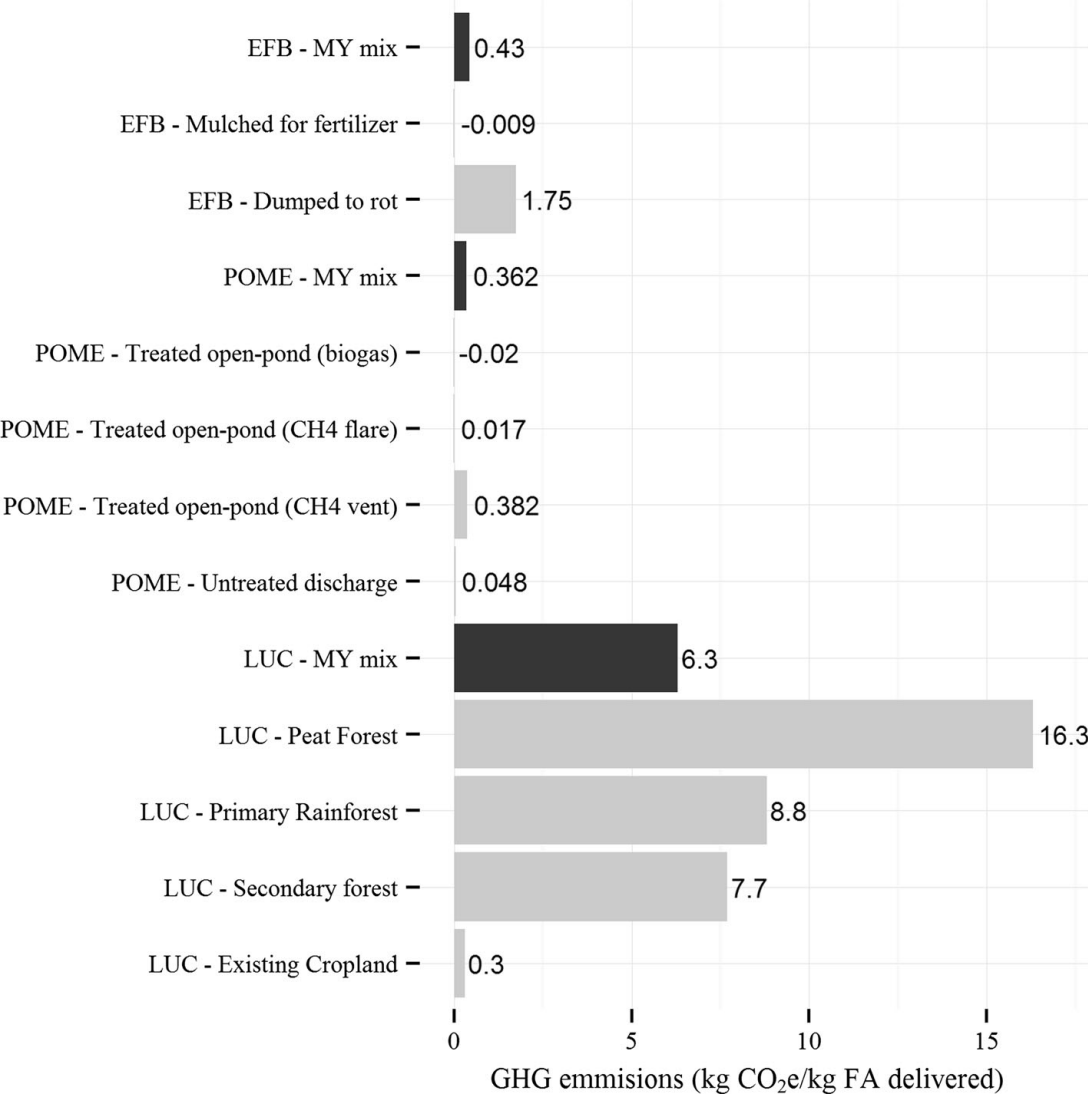
Results of various sensitivity analyses, namely, land use change (LUC), POME (wastewater effluent from palm oil mill) treatment, and EFB (empty fruit bunches) treatment, are shown in kg CO_2_e/kg FA delivered. The base case MY mix GHG emissions represent the typical practices for palm plantations in Malaysia (MY). For LUC, the practices for the base case are 13 % LUC from peat forest, 52 % from secondary forest and rest 35 % from existing cropland. Peat forest has the most GHG emissions, while they are the least for the transformation of existing cropland with carbon debt paid off. For POME treatment, the practices for the base case are 5 % of POME being used for generation of biogas for heat production and the rest 95 % being treated emitting the resulting biogas. The venting of biogas from anaerobic treatment has the most GHG emissions, while the anaerobic treatment with the resulting methane recovered and utilized for heat generation has the least. For EFB treatment, the practices for the base case are 75 % of EFB mulched and rest 25 % dumped to rot. Mulching of EFB for a fertilizer substitute shows the least life cycle GHG emissions, while the dumping of EFB to rot has the most

The contribution analyses for climate change suggest that land use change, POME treatment and EFB treatment are critical factors for life cycle GHG emissions from PKO-FA production. The results of sensitivity analyses for these three key parameters are summarized in Fig. 4. EFB could be mulched and used as fertilizer or dumped to rot. In the latter case, methane, carbon dioxide and nitrous oxide could be emitted depending on the anaerobic conditions. This translates into mulching of EFB for fertilizer being a better option. Among the evaluated POME end-of-life treatment options, anaerobic treatment with the resulting methane recovered and utilized for heat generation has the least life cycle GHG emissions. The venting of methane from anaerobic treatment has the most GHG emissions, even higher than discharging untreated POME. When LUC options are considered, GHG emissions are the highest when peat forests are transformed for palm cultivation and the lowest when existing croplands (whose carbon debt has been paid off)^5^ are transformed. The sensitivity analyses show that PKO-FA has lower GHG emissions with petro-FA from an environmental perspective if the existing cropland is used for palm plantation instead of land transformation. Further, in such scenario, CO_2_ could be sequestered compared to petro-FA. In an ideal situation when PKO is entirely produced on existing cropland, POME is being treated with methane recovered for thermal energy generation and EFB is used for mulching to replace some fertilizer needs, PKO-FA have GHG emissions of approximately −1.5 kg CO_2_e/kg FA delivered, thereby outperforming petro-FA. However, if 100 % of PKO comes from peatlands drainage and deforestation, POME is treated with recovered methane vented, and EFB is dumped to rot under anaerobic conditions, the GHG emissions increase to ~16.7 kg CO_2_e/kg FA delivered.

Among the other impact categories, PKO-FA has less metal depletion, less fossil depletion, less human toxicity, less ionizing radiation emissions, less metal depletion, less ozone depletion and less water depletion on average (see Table 2). While LUC affects most other impact categories (except terrestrial ecotoxicity and agricultural land occupation), among them natural land transformation, marine eutrophication, particulate matter formation and photochemical oxidant formation see significant effects. Urban land occupation and water depletion are also affected. While GHG emissions for discharging of POME without treatment is not significant, the impacts on eutrophication from this option is ~100 times more than other options. Besides impacts on climate change and eutrophication, the POME treatment options also affect terrestrial ecotoxicity, particulate matter formation, photochemical oxidant formation, human toxicity and terrestrial acidification. The treatment of EFB impacts all impact categories as all of them show a positive environmental profile for mulching compared to burden for all impact categories when dumped to rot.

**Table 2:** Comparing mean values (and coefficient of variation) results of all impact categories

Impact category	Unit	Petro FA		PKO FA	
		Average	SD	Average	SD
Agricultural land occupation	m^2^a	4.25E-02	3.17E-02	1.82E+ 00	6.12E-01
Climate change	kg CO_2_ eq	2.97E+00	5.23E-01	5.27E+00	4.57E+00
Fossil depletion	kg oil eq	1.84E+00	3.93E-01	5.99E-01	1.30E+00
Freshwater ecotoxicity	kg 1,4-DB eq	2.14E-02	2.09E-01	4.16E-02	6.08E-01
Freshwater eutrophication	kg P eq	4.58E-04	3.03E-04	6.29E-04	1.85E-03
Human toxicity	kg 1,4-DB eq	−7.75E-02	5.64E+01	−9.19E+00	3.21E+02
Ionizing radiation	kBq U235 eq	1.56E-01	1.84E-01	8.78E-02	4.37E-01
Marine ecotoxicity	kg 1,4-DB eq	1.78E-02	1.69E-01	1.95E-02	4.18E-01
Marine eutrophication	kg N eq	3.29E-04	6.92E-05	1.30E-02	4.39E-03
Metal depletion	kg Fe eq	1.31E-01	8.07E-02	1.22E-01	8.87E-01
Natural land transformation	m^2^	2.02E-04	1.26E-04	3.49E-02	1.14E-02
Ozone depletion	kg CFC-11 eq	1.02E-07	4.67E-08	5.86E-08	3.60E-07
Particulate matter formation	kg PM_10_ eq	3.70E-03	7.72E-04	8.59E-03	9.55E-03
Photochemical oxidant formation	kg NMVOC	1.32E-02	3.28E-03	1.56E-02	2.34E-02
Terrestrial acidification	kg SO_2_ eq	1.22E-02	3.11E-03	1.79E-02	2.29E-02
Terrestrial ecotoxicity	kg 1,4-DB eq	1.03E-04	1.45E-03	1.39E-01	3.95E+00
Urban land occupation	m^2^a	1.04E-02	3.37E-03	2.42E-02	2.80E-01
Water depletion	m^3^	2.71E+00	5.27E-01	1.37E+00	7.25E+00

*SD* standard deviation

**Fig. 5:**
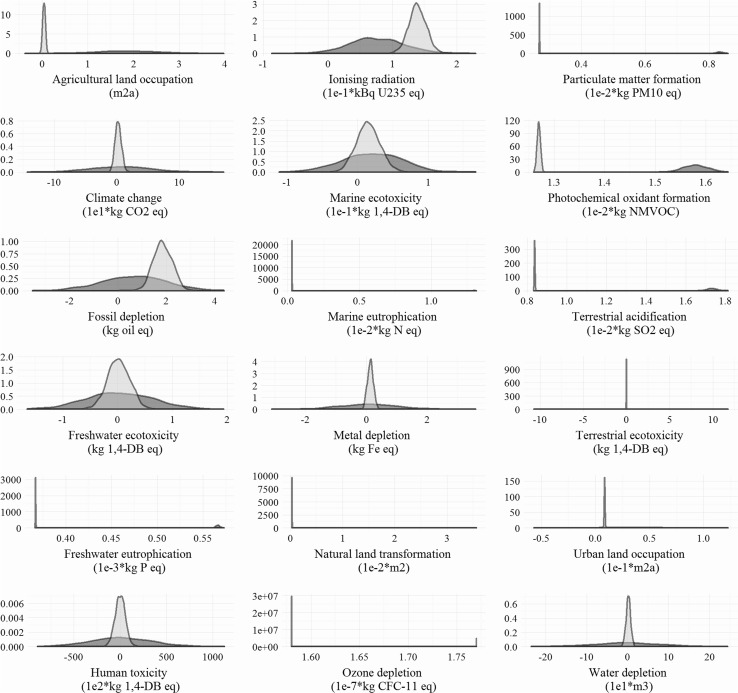
Results of uncertainty analyses (1000 runs of Monte Carlo using the in-built function in Simapro 8.0) for characterized impacts for PKO-FA (fatty alcohol produced from palm kernel oil feedstock) and petro-FA (fatty alcohol produced from petrochemical feedstock) are presented for all 18 evaluated impact categories as density plots. For PKO-FA, the distributions of impacts for all impact categories are broader compared to the narrow distribution for petro-FA. Marine eutrophication, agricultural land occupation, natural land occupation, fossil depletion, particulate matter formation, water depletion and climate change have the largest difference in the impacts between petro-FA and PKO-FA

**Fig. 6:**
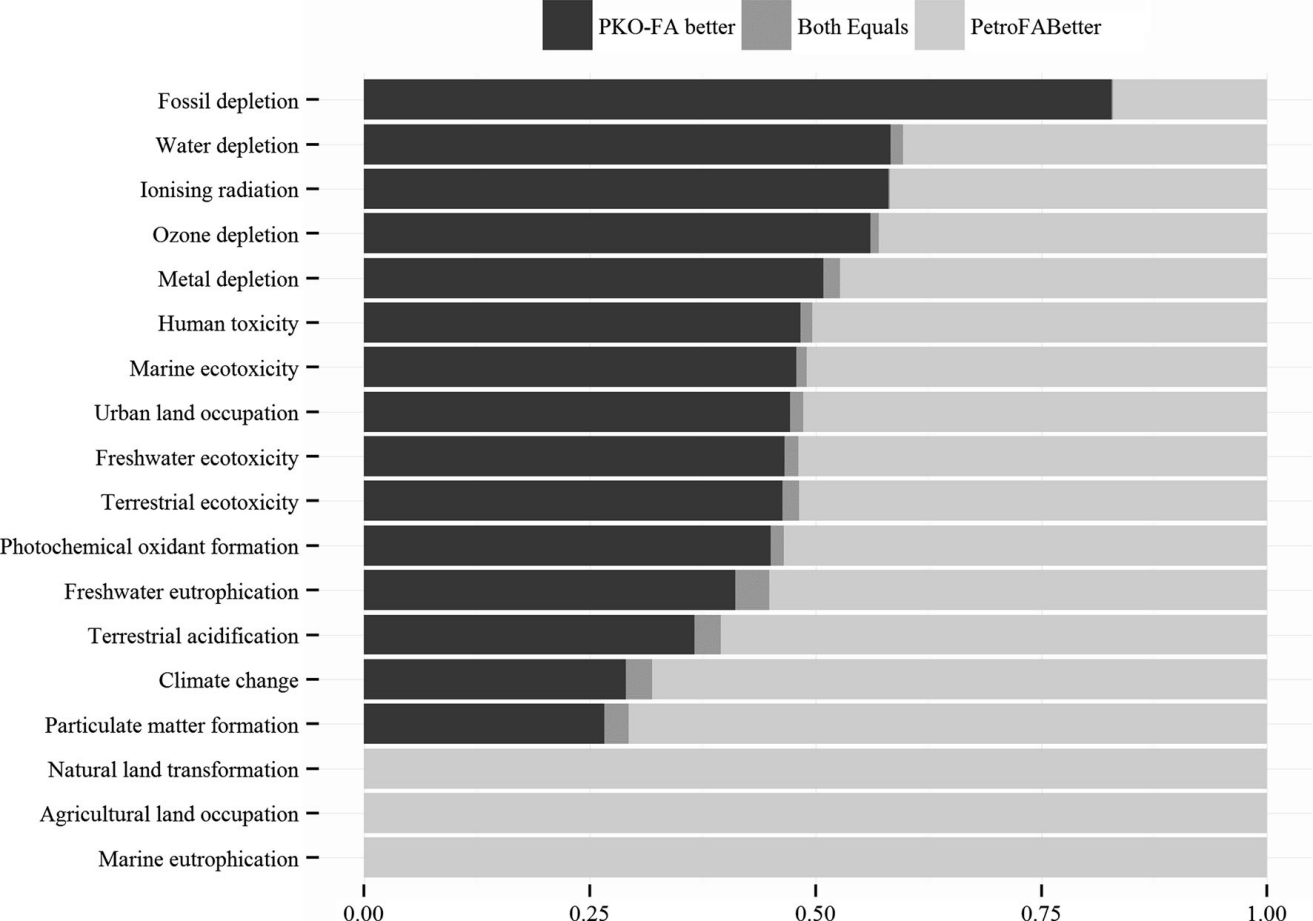
Results of uncertainty analyses (1000 runs of Monte Carlo using the in-built function in Simapro 8.0) for characterized impacts for PKO-FA (fatty alcohol produced from palm kernel oil feedstock) and petro-FA (fatty alcohol produced from petrochemical feedstock) are presented for all 18 impact categories as a percentage of the samplings for which a particular option had lower impacts. For example, petro-FA has lower or equal GHG emissions for ~70 % of samplings and PKO-FA causes lower or equal water depletion for ~60 % of samplings

The uncertainty analyses were performed to obtain the distribution of the environmental impacts for both petro-FA and PKO-FA. The results for all 18 evaluated impact categories have been captured in Fig. 5 via density plots. In these density plots, the broader distribution for an impact category represents higher uncertainty. For PKO-FA, the distributions of impacts for all impact categories are broader compared to the narrow distribution for petro-FA. The higher uncertainty for PKO-FA is from the variations in the practices with palm plantations and oil (palm oil and PKO) production processes. Further, the higher overlap area for an impact category in density plots represents a lower difference between the compared options. Marine eutrophication, agricultural land occupation, natural land occupation, fossil depletion, particulate matter formation, water depletion and climate change have the least overlapped area and, hence, have the largest difference in the impacts between petro-FA and PKO-FA. The extent of overlap in distribution can also be represented as the percentage of samplings for which a particular option had lower impacts. For example, petro-FA has lower or equal GHG emissions for ~70 % of samplings and PKO-FA causes lower or equal water depletion for ~60 % of samplings. Figure 6 summarizes the results of such representation for PKO-FA being better and/or equal to petro-FA for all 18 impact categories.

## Discussion

**Fig. 7:**
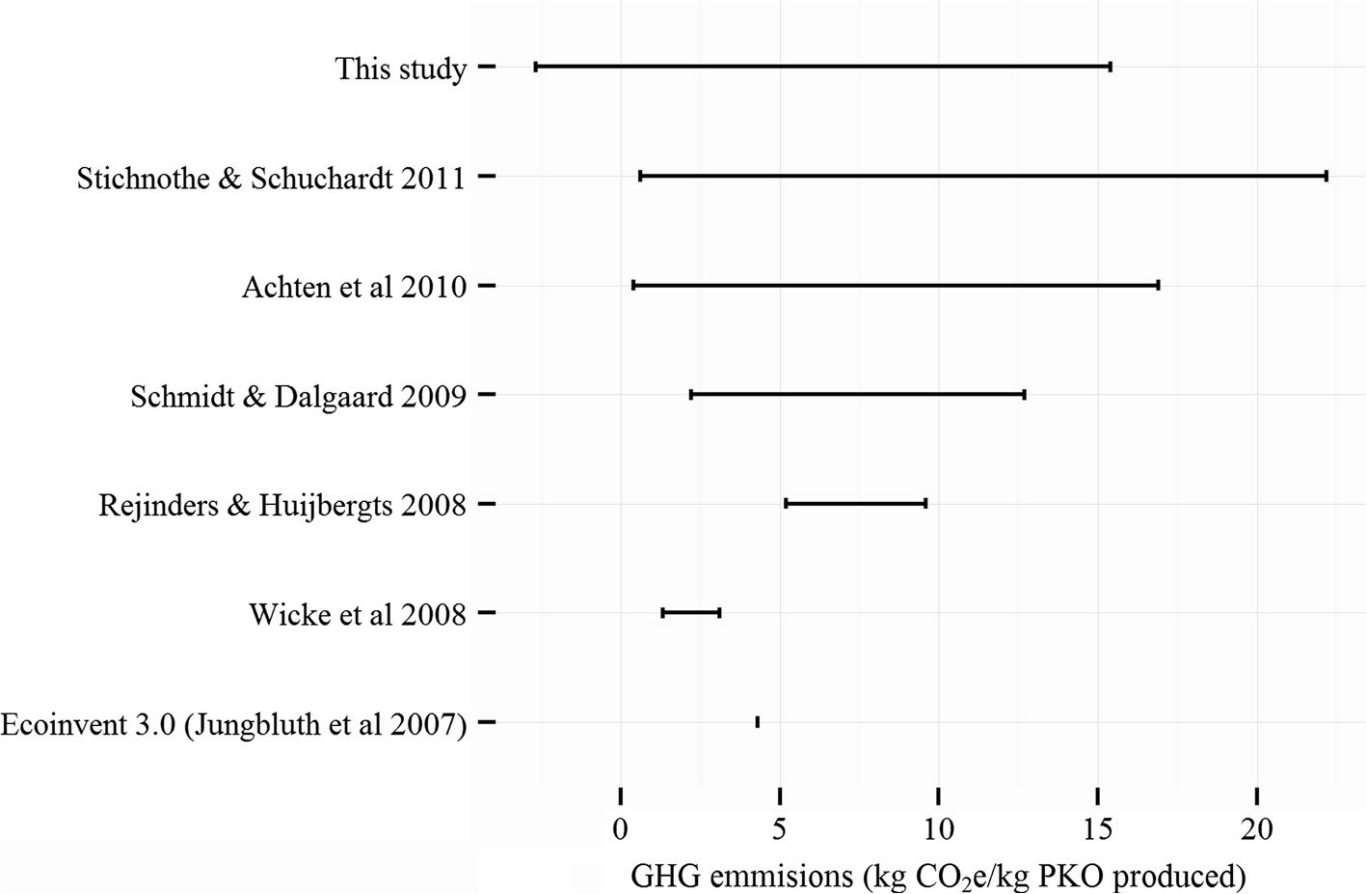
Literature data on the life cycle GHG (greenhouse gas) emissions for oil produced from Palm fruit in kg CO_2_e/kg oil produced. Depending on the operating practices, the GHG emissions as per this LCA study varies from −2.7 to 15.4 kg CO_2_e/kg oil produced. Such significant variances in the GHG emissions for PKO-FA were also observed by Stichnothe and Schuchardt [10] (0.6–22.2 kg CO_2_e/kg oil produced), Achten et al. (0.4–16.9 kg CO_2_e/kg oil produced) [17] and Schmidt and Dalgaard [29] (2.2–12.7 kg CO_2_e/kg oil produced). While the variances observed by Rejinders and Huijbergts [25] (5.2–9.6 kg CO_2_e/kg oil produced) and Wicke et al. [21] (1.3–3.1 kg CO_2_e/kg oil produced) were not equally large, their ranges are within those observed. The potential emissions estimated by Jungbluth et al. [11], as part of EcoInvent 3.0 dataset, also falls within the observed ranges

Both the petrochemical and PKO feedstocks being part of large and complex supply chains is expected and documented in the literature [6, 8]. Our GHG emissions results are in alignment with the literature evaluating the similar claims for palm oil (PO) for other fossil resource substitutions. While on average PKO-FA performs worse, life cycle GHG emissions for PKO-FA could be lower than those for petro-FA under limited conditions as per sensitivity analyses. Such significant variances in the GHG emissions for PKO-FA (observed from uncertainty analyses and sensitivity analyses) are in accordance with the results of previous studies [10, 11, 41, 45, 51, 52] summarized in Fig. 7. These variances are expected due to the variances in agricultural and forestry practices such as fertilizer applications, pesticides applications, properties of soil, growth rate (and, hence CO_2_ absorption) for the plants and handling of biomass and co-products. Hence, the environmental friendliness of PKO-FA for GHG emissions reduction varies with the actual practice, which is in consensus with findings by Reijnders and Huijbergts [45]. Land use change, POME end-of-life treatments and EFB end-of-life treatments are key parameters, which were also observed in previous studies [10, 45].

The selection of raw material sourcing for FA production involves trade-offs as PKO-FA performs better on average in six impact categories while petro-FA performs better on average in another 12 impact categories. Such trade-offs have been observed by Stalmans et al. [6] and are expected due to inherent differences between the bio-based value chain and the fossil-based value chain. Marine eutrophication, agricultural land occupation, natural land occupation, fossil depletion, particulate matter formation, water depletion and climate change are key impact categories for the considered FA sourcing options as shown in Table 3.

**Table 3:** List of processes that are major contributors for the identified impact categories for both petro-FA and PKO-FA

	Petro-FA	PKO-FA
Climate change	*N*-Olefin Ethylene Fuel combustion (process) Aluminum powder Chemical plant (<5 %)	Land use change for palm plantation Palm plantation operation Fuel combustion emissions (transportation)
Fossil fuel depletion	*N*-Olefin Ethylene Fatty alcohol production (<5 %) Fuel combustion (process) Aluminum powder (<5 %)	Fuel combustion (process, transportation**)** Electricity production
Natural land transformation	Oil extraction for *n*-olefins, ethylene and fuel for transportation NG extraction for fuel for process Chemical plants Aluminum powder	Land use change for palm plantation Land use change for oil extraction (<0.3 %)
Eutrophication	Sulfidic tailings for production of Copper used in chemical plants Spoil from mining of coal used for electricity production Production of Gold used in electronics for chemical plants Treatment of dross from electrolysis for Copper and Aluminum production Incineration of solid waste	Fertilizer use for palm plantations Irrigation for palm plantations Sulfidic tailings for production of Copper used in chemical plants Spoil from mining of coal used for electricity production
Agricultural land occupation	Wood used for building material in chemical plants (<0.1 % of impacts for PKO-FA)	99+% from palm plantations
Water depletion	Aluminum powder (~50 %) *N*-olefin Chemical plant Electricity used during FA production	Palm FFB growth Fuel combustion (process, transportation**)** Chemical plant Electricity used during FA production
Particulate matter formation	*N*-Olefin Ethylene Fuel combustion (process) Aluminum powder Electricity used during FA production	Palm FFB growth Fuel combustion (process, transportation**)**

Our findings must be interpreted in accordance with the scope of this study and the limitations due to the use of secondary data and assumptions. Further, this LCA study does not evaluate the implications of shifting to one particular feedstock, which could affect the inefficiencies and efficiencies of the individual systems. The overall larger systems to which each feedstock belongs should also be considered along with sustainability values of the specific stakeholders, the socio-economic relevance and other aspects not covered. Besides the feedstocks themselves being derived through multi-output processes, both petro-FA and PKO-FA are multi-output processes. Currently, the environmental impacts are allocated from the processes to the co-products. The changes in economics for the co-products through supply and demand dynamics will influence how the co-products are handled and, hence, the environmental impacts.

Currently, there is increasing demand for the bio-derived products due to their perceived environmental benefits. The results show that the environmental impacts for PKO-FA strongly depend on palm plantations and palm oil mill operation practices. Hence, we recommend being mindful of the upstream practices specific to the suppliers when sourcing bio-derived materials. With the adoption of proper practices including decisions on land use changes, the bio-derived materials such as PKO provide a good environmentally friendly alternative to the non-renewable raw materials. While PKO and such bio-derived materials provide renewability in terms of carbon recycling and regenerating through cultivation, the responsibly produced bio-derived materials are limited by the availability of suitable land. Similar to the other renewable resources there are limits for environmentally responsible harvesting for PKO. The results of this LCA study show that petro-FA has a better average life cycle environmental performance than PKO-FA for the majority of environmental impact categories we investigated. This highlights that environmentally responsible sourcing should require rigorous testing of the assumption of “automatic environmental benefits” for bio-derived raw materials. Also, the intrinsic sustainability values of the stakeholders based on the respective local environmental profiles would be critical in incorporating the trade-offs into decision making.

## Electronic supplementary material

Below is the link to the electronic supplementary material.

Supplementary material 1 (DOCX 75 kb)

## Abbreviations

AE: Alcohol ethoxylates
AGB: Above ground biomass
BGB: Below ground biomass
Ca: Calcium
CO_2_e: Carbon dioxide equivalent of global warming potential
COD: Chemical oxygen demand
DOM: Dead organic matter
EFB: Empty palm fruit bunches
EI3.0: EcoInvent v3.0 database
EO: Ethylene oxide
EPA: US Environmental Protection Agency
FA: Fatty alcohol
FFB: Fresh palm fruit bunches
GHG: Greenhouse gases
GLO: Global
K: Potassium
K_2_O: Potassium oxide
LCA: Life cycle assessment
LCI: Life cycle inventory
LCIA: Life cycle impact assessment
LUC: Land use change
Mg: Magnesium
MY: Malaysia
N: Nitrogen
NP: Nonylphenol
NPEs: Nonylphenol ethoxylates
Oleo-FA: Fatty alcohol produced from oleochemical feedstock
P: Phosphorus
P_2_O_5_: Phosphorus pentaoxide
Petro-FA: Fatty alcohol produced from petrochemical feedstock
PKE: Palm kernel extract
PKO: Palm kernel oil
PKO-FA: Fatty alcohol produced from palm kernel oil as feedstock
PKS: Palm kernel shells
PO: Palm oil
POME: Palm oil mill effluent
RoW: Rest of world
SERC: Southeastern Electric Reliability Council
US: United States of America

## Footnotes

1 The feedstock for oleo-FA can be either PKO or coconut oil. For this study, the scope has been restricted to PKO feedstocks.

2 While Rhodium catalyst are used for ~75 % of hydroformylation processes [21], Cobalt catalysts are used for hydroformylation of higher olefins for detergent end-use alcohols [21, 22].

3 BP exited the business [25].

4 When the function of the land before LUC still needs to be met, a LUC might lead to additional LUC due to shifting of the current function to another location. Such additional LUC is classified as indirect LUC.

5 While these results could be interpreted as a need for a policy of sourcing from no land use change PKO so as to allow sourcing FA with lower footprint, the authors of this study would like to caution against such interpretation based on these results. This study does not account for indirect LUC, which could be happening. Also, such policy might only increase demand for such non-LUC-PKO; thereby, shifting land use change to other human activities.
