# Supplementary material for: Comparison of Oleo- vs Petro-Sourcing of Fatty Alcohols via Cradle-to-Gate Life Cycle Assessment
Source: J Surfactants Deterg. 2016 Sep 12;19(6):1333–51. doi: 10.1007/s11743-016-1867-y (PMC5055910; doi:10.1007/s11743-016-1867-y)
Supplement: Supplementary file 1 — Supplementary material 1 (DOCX 75 kb) [file 11743_2016_1867_MOESM1_ESM.docx]

Comparison of Oleo- vs Petro- sourcing of Fatty Alcohols via Cradle-to-gate Life Cycle Assessment – Supplementary file

# Authors

Jignesh Shah*, Sustainability Engineer, Air Products and Chemicals, Inc., 7201 Hamilton Blvd, Allentown, PA 18195, shahjj@airproducts.com, 610-418-8712

Erdem Arslan, Senior Principal Systems Engineer, Air Products and Chemicals, Inc., 7201 Hamilton Blvd, Allentown, PA 18195

John Cirucci, Senior Engineering Associate, Air Products and Chemicals, Inc., 7201 Hamilton Blvd, Allentown, PA 18195

Dave Moss, Americas Technology Manager, Versum Materials, Inc.., 7201 Hamilton Blvd, Allentown, PA 18195

Julie O’Brien, Corporate Sustainability Director, Air Products and Chemicals, Inc., 7201 Hamilton Blvd, Allentown, PA 18195

# Biography


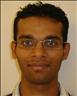
Jignesh Shah is a sustainability engineer at Air Products and Chemicals, Inc. After completing B.S. in Chemical and Biomolecular Engineering at Georgia Institute of Technology, he joined Air Products and Chemicals, Inc. as a chemical engineer in 2007 and has been enjoying his current role for past 2+ years. He has realized his passion in sustainability assessments through achieving M.S. in Sustainability Engineering at Villanova University in 2013 and through 10 life cycle studies in diverse topics. Currently, he is dreaming (ha!) about combining two of his passions – process engineering and sustainability.

# Abbreviations

| AE | Alcohol Ethoxylates |
| --- | --- |
| AGB | Above Ground Biomass |
| BGB | Below Ground Biomass |
| Ca | Calcium |
| CO_2_e | Carbon dioxide equivalent of global warming potential |
| COD | Chemical Oxygen Demand |
| DOM | Dead Organic Matter |
| EFB | Empty Palm Fruit Bunches |
| EI3.0 | EcoInvent v3.0 database |
| EO | Ethylene Oxide |
| EPA | U.S. Environmental Protection Agency |
| FA | Fatty Alcohol |
| FFB | Fresh Palm Fruit Bunches |
| GHG | Greenhouse gases |
| GLO | Global |
| K | Potassium |
| K_2_O | Potassium oxide |
| LCA | Life Cycle Assessment |
| LCI | Life Cycle Inventory |
| LCIA | Life Cycle Impact Assessment |
| LUC | Land Use Change |
| Mg | Magnesium |
| MY | Malaysia |
| N | Nitrogen |
| NP | Nonylphenol |
| NPEs | Nonylphenol Ethoxylates |
| Oleo-FA | Fatty alcohol produced from oleochemical feedstock |
| P | Phosphorus |
| P_2_O_5_ | Phosphorus Pentaoxide |
| Petro-FA | Fatty alcohol produced from petrochemical feedstock |
| PKE | Palm Kernel Extract |
| PKO | Palm Kernel Oil |
| PKO-FA | Fatty alcohol produced from palm kernel oil as feedstock |
| PKS | Palm Kernel Shells |
| PO | Palm Oil |
| POME | Palm Oil Mill Effluent |
| RoW | Rest of World |
| SERC | Southeastern Electric Reliability Council |
| US | United States of America |

This document is supplementary file that summarizes the data used for generating LCI for upstream process for PKO production. The impacts from the various end-of-life treatment options for POME can be found in Table S1. The adoption rate of these treatment options in Malaysia shown in Table S2. The initial nutrient values for EFB are shown in Table S3. These data were used to estimate emissions to air and soil from dumping of EFB. For mulching, the fertilizer value of the mulch was estimated based on literature data shown in Table S4. The adjustments for the LUC not covered in the datasets in SimaPro 8.0 were based on the GHG emissions (tonne CO2e/hectare) for deforestation / land transformation shown in Table S5.

Table S1 Inputs for inventory of impacts from various Palm Oil Mill Effluent treatment options [1]

| Parameter | Description | River discharge | Average | Standard Deviation |
| --- | --- | --- | --- | --- |
| COD_POME_ (kg COD/m^3^ POME) | Chemical Oxygen Demand generated from discharge of organics in POME | Untreated | 25.41 | 2.5 |
|  |  | Treated | 0.150 | 0.025 |
| B_0_ | Methane producing capacity from the organics discharged |  | 0.23 | 0.01 |
| CF_CH4_ | Correction factor to the methane production capacity based on the conditions into which organics are discharged | Untreated | 0.1 | 0.03 |
|  |  | Treated in anaerobic digestor/pond | 0.8 | 0.05 |
| Ncontent_POME_ (kg N/m^3^ POME) | Nitrogen content discharge in the river | Untreated | 0.150 | 0.025 |
|  |  | Treated | 0.080 | 0.010 |

Table S2 POME treatment methods currently practiced in Malaysia [2]

| POME end-of-life option | Relevance in Malaysia |
| --- | --- |
| Untreated discharge to river | 0% |
| Anaerobic digestion w/ CH4 venting | 95% |
| Anaerobic digestion w/CH4 flaring | 0% |
| Anaerobic digestion w/ CH4 for heat generation | 5% |

Table S3 EFB composition dry mass basis (used to estimate emissions to air and soil from dumping)

| Nutrients | Average for this study | Standard Deviation | Conversion factors |
| --- | --- | --- | --- |
| Nitrogen (N) | 0.60% [3*,5**,6] | 0.0023 |  |
| Phosphorus (P) | 0.08% [3*,5**,6] | 0.00043 | 43.6% P in P_2_O_5_ |
| Potassium (K) | 2.18% [3*,5**,6] | 0.00634 | 83% K in K_2_O |
| Calcium (Ca) | 0.27% [3*,6] | 0.00141 | 71.5% Ca in CaO |
| Magnesium (Mg) | 0.21% [3*,5**,6] | 0.00071 | 60.3% Mg in MgO |
| Carbon (C) | 65% [3*] | 0.00273 |  |
| *Includes upper range and lower range, **includes data from four studies | | | |

Table S4 Fertilizer value (kg) for mulching 1 tonne of EFB

|  | Average for this study | Standard Deviation | Conversion factors |
| --- | --- | --- | --- |
| Urea eq | 6.06 [3,4,5*,6,7] | 1.658 | 46% N in Urea, 34% N in Ammonium nitrate |
| P_2_O_5_ eq | 0.92 [3,4,5*,6,7] | 0.540 | 30%-35% P_2_O_5_ in Phosphate rocks, 43.6% P in P_2_O_5_ |
| Potash Muriate | 19.31 [3, 5*,6,7] | 2.007 | 50% of Potash Muriate is K |
| Keiserite | 6.05 [3, 5*,6] | 2.727 |  |
| *65% water in EFB [3,4,6,7] | | | |

Table S5 GHG emissions (tonne CO_2_e/hectare) for deforestation / land transformation from these original land types

|  | Peatland | Primary Forest | Secondary Forest | Existing cropland |
| --- | --- | --- | --- | --- |
| Malaysia | ~44* [5,8] | 216 [9] | Assume same proportional to Primary forest as for other regions |  |
| India |  | 173 [9] | 128 [9] |  |
| Rest of World |  | 185 [9] | 137 [9] |  |
| Land used for Palm Plantation in MY | 13% [2] | 0% | 52% [2, 9] | 35% [9] |
| * Additional to primary forest due to BGB | | | | |

# Funding

This study was funded in its entirety by Air Products and Chemicals, Inc. The third party critical review by Intertek was funded by Air Products and Chemicals, Inc.

# References

1. Deru M, Torcellini P (2007) Source Energy and Emission Factors for Energy Use in Buildings. National Renewable Energy Laboratory. NREL/TP-550-38617. http://www.nrel.gov/docs/fy07osti/38617.pdf
2. Noweck K, Grafahrend W (2012) Fatty Alcohols. In: Ullmann's Encyclopedia of Industrial Chemistry Vol 14. Wiley-VCH, Weinheim. DOI: 10.1002/14356007.a10_277.pub2
3. Alibaba (accessed Jan 2015) Industrial Metal Powder Aluminum Powder Production Line. http://www.alibaba.com/product-detail/Industrial-Metal-Powder-Aluminum-Powder-Production_447115456.html
4. Serresa N, Tidua D, Sankareb S, Hlawkaa F (2011) Environmental comparison of MESO-CLAD® process and conventional machining implementing life cycle assessment. Journal of Cleaner Production 19 (2011) 1117-1124. DOI:10.1016/j.jclepro.2010.12.010
5. LSU Chemistry Homepage (accessed Jan 2015) Hydroformylation (Oxo) Catalysts. http://chem-faculty.lsu.edu/stanley/webpub/4571-Notes/chap16-Hydroformylation.docx
6. Lloyd, L (2011) Handbook of Industrial Catalyst. In: Twigg MV, Spencer MS (eds) Fundamental and Applied Catalysis. Springer New York Dordrecht Heidleberg London DOI: 10.1007/978-0-397-49962-8
7. Gankin VY, Gurevich GS. Chemical Technology of Oxosynthesis. Institute of Theoretical Chemistry: Editorial Board 'Khimiya' ('Chemistry'), St. Petersburg. http://en.itchem.ru/d/216737/d/chemical_technology_of_oxosyntheisi.pdf
8. ICIS Chemical Business (accessed Jan 2015) Fatty Alcohols Market Reels from Oversupply, Weak Demand. http://www.icis.com/resources/news/2002/10/28/183696/fatty-alcohols-market-reels-from-oversupply-weak-demand/
9. Jungbluth N, Chudacoff M, Duariat A, Dinkel F, Doka G, Faist Emmenegger M, Gnansounou E, Kljun N, Schleiss K, Spielmann M, Stettler, C, Sutter J (2007) Life Cycle Inventories of Bioenergy. Ecoinvent report No. 17, Swiss Centre for Life Cycle Inventories, Dübendorf, CH.
